# Case Report: ^18^F-FDG PET/CT for precision assessment of tumor aggressiveness in canine subcutaneous leiomyosarcoma

**DOI:** 10.3389/fvets.2026.1948200

**Published:** 2026-10-19

**Authors:** Yeon Chae, Dongseop Lee, Yoonhoi Koo, Taesik Yun, Hakhyun Kim, Byeong-Teck Kang

**Affiliations:** 1 Laboratory of Veterinary Internal Medicine, College of Veterinary Medicine, Chungbuk National University, Cheongju, Chungbuk, Republic of Korea; 2 College of Veterinary Medicine, Kyungpook National University, Daegu, Republic of Korea

**Keywords:** diagnostic imaging, soft tissue sarcoma, subcutaneous leiomyosarcoma, tumor grading, veterinary oncology, ^18^F-FDG PET/CT

## Abstract

A 9-year-old intact male Alaskan Malamute was presented with a hemorrhagic, ulcerative skin lesion on the right elbow. Cytological examination revealed bacterial infection and atypical epithelioid-appearing cells exhibiting anisocytosis and anisokaryosis; however, mesenchymal neoplastic cells were not identified. Despite antimicrobial therapy, the lesion progressed rapidly. Histopathological examination of the biopsy specimen was consistent with a grade 2 soft tissue sarcoma; however, the clinical behavior was more aggressive than anticipated for this grade, prompting further evaluation. 18F-fluorodeoxyglucose (^18^F-FDG) positron emission tomography/computed tomography (PET/CT) demonstrated high metabolic activity of the mass, with a maximum standardized uptake value of 10.1 and mean standardized uptake value of 4.6, with no evidence of distant metastasis. Surgical resection was performed about 2 weeks after PET/CT. Notably, histopathological examination of the surgical specimen revealed a grade 3 poorly differentiated mesenchymal neoplasm with incomplete surgical margins, indicating that the preoperative biopsy had underestimated the final tumor grade and that the high ^18^F-FDG avidity observed on PET/CT was more consistent with the final histopathologic grade. Immunohistochemical staining confirmed the diagnosis of subcutaneous leiomyosarcoma. Although adjuvant chemotherapy was recommended, only palliative therapy was provided at the owner’s request. Local recurrence and pulmonary lesions suspected to be metastatic developed 3 months postoperatively, and the dog expired 6 months after surgery. To the best of our knowledge, this is the first report of ^18^F-FDG PET/CT findings in canine subcutaneous leiomyosarcoma, underscoring the utility of this imaging modality in evaluating tumor aggressiveness and guiding treatment planning.

## 1 Introduction

Leiomyosarcoma (LMS) is a malignant tumor originating from smooth muscle cells. It is reported to occur mainly in visceral organs, but also reported infrequently in non-visceral locations such as eyelid, limbs, penis, and skin (1–3). While subcutaneous LMS is sometimes considered a subtype of soft tissue sarcoma (STS) (4), studies suggest that it should be independently classified due to its distinct biological behavior and anatomical location (2, 4, 5). Diagnosis of LMS and differentiation from other subtypes rely on biopsy and histopathologic evaluation, and immunohistochemical analysis, which are necessary for treatment planning and prognosis prediction (2, 4). However, due to its rarity in subcutaneous tissues, the biological behavior and prognosis of non-visceral LMS remain poorly understood (2, 4). Furthermore, preoperative biopsy of STS may not always reflect true tumor grade due to intratumoral heterogeneity (6), potentially leading to underestimation of malignancy and suboptimal treatment planning.

18F-fluorodeoxyglucose (^18^F-FDG) positron emission tomography/computed tomography (PET/CT) is a nuclear imaging modality widely used in oncology for tumor detection, staging, and therapeutic monitoring (7). By assessing tissue metabolic activity, PET/CT can provide functional insights that complement conventional imaging techniques, potentially predicting tumor behavior and aggressiveness. Various studies have reported the utility of ^18^F-FDG PET/CT in human STS and LMS in the diagnosis and clinical staging (7–11). However, its application in veterinary oncology remains limited, with only a few studies characterizing FDG uptake in canine STS (12), and no reports describing PET/CT findings in canine subcutaneous LMS to date.

This case report describes the first ^18^F-FDG PET/CT findings in canine subcutaneous LMS and aims to highlight the utility of this imaging modality in evaluating tumor aggressiveness, particularly in cases where preoperative histopathological assessment may underestimate malignancy.

## 2 Case description

A 9-year-old, 40 kg intact male Alaskan Malamute presented with a hemorrhagic and ulcerative skin lesion over the caudal aspect of the right elbow, which had first been noticed by the owner 3 weeks before presentation and had remained static in size and appearance (Figure 1a). A timeline of the clinical course is provided in Supplementary Table S1. Based on the clinical appearance, infectious, inflammatory, and neoplastic etiologies were considered. Radiographs of the right elbow revealed a 4.3 × 3.1 cm oval soft tissue mass over the caudal aspect of the right elbow joint with a relatively radiolucent center, without evidence of adjacent cortical bone lysis (Supplementary Figure S1a). To investigate the underlying causes, an impression smear and fine needle aspiration were performed. The smear revealed moderate numbers of cocci and rods with degenerate neutrophils and hemorrhage, while fine needle aspiration revealed cohesive clusters of atypical cells presumed to be of epithelial origin, exhibiting marked anisocytosis, anisokaryosis, and prominent nucleoli. Mesenchymal neoplastic cells were not identified in the FNA sample. Given the ulceration and inflammation of the lesion, the atypical epithelioid-appearing cells may have represented reactive or dysplastic epithelial changes associated with the ulcerated lesion rather than the underlying neoplastic cell population. Therefore, biopsy for histopathologic examination was recommended to establish a definitive diagnosis. As the owner declined invasive diagnostic procedure, empirical treatment targeting both bacterial and possible fungal pathogens was initiated with oral amoxicillin-clavulanate, itraconazole, topical 0.5% chlorhexidine solution, and 1% silver sulfadiazine cream. Despite 9 days of treatment targeting infection, the lesion showed no improvement, and rapidly enlarged into a distinct mass arising from the surface of the elbow (Figure 1b). To differentiate between benign and malignant skin disease, biopsy was performed for histopathological evaluation. The histopathology revealed neoplastic tissues within the subcutaneous tissue and fascicles of spindle-shaped cells with pleomorphic nuclei, consistent with a diagnosis of STS (differentiation score 2), with fibrosarcoma or LMS considered as possible subtypes (Figure 2a). The mitotic index was 5 per 10 high-power fields (mitotic score 1) and necrosis was present in less than 50% of the sample (necrosis score 2). Using the three-tiered grading system for canine STS (13), the summed score of 5 corresponded to a grade 2 STS. The lesion progressed more aggressively than typically expected for a grade 2 STS. Moreover, the possibility of skin metastasis could not be ruled out. Therefore, further evaluation was required to determine the primary site and assess potential metastasis before establishing an appropriate treatment plan. Consequently, ^18^F-FDG PET/CT was performed 17 days after biopsy to evaluate the metabolic activity of tumor and possible metastasis.

**Figure 1:**
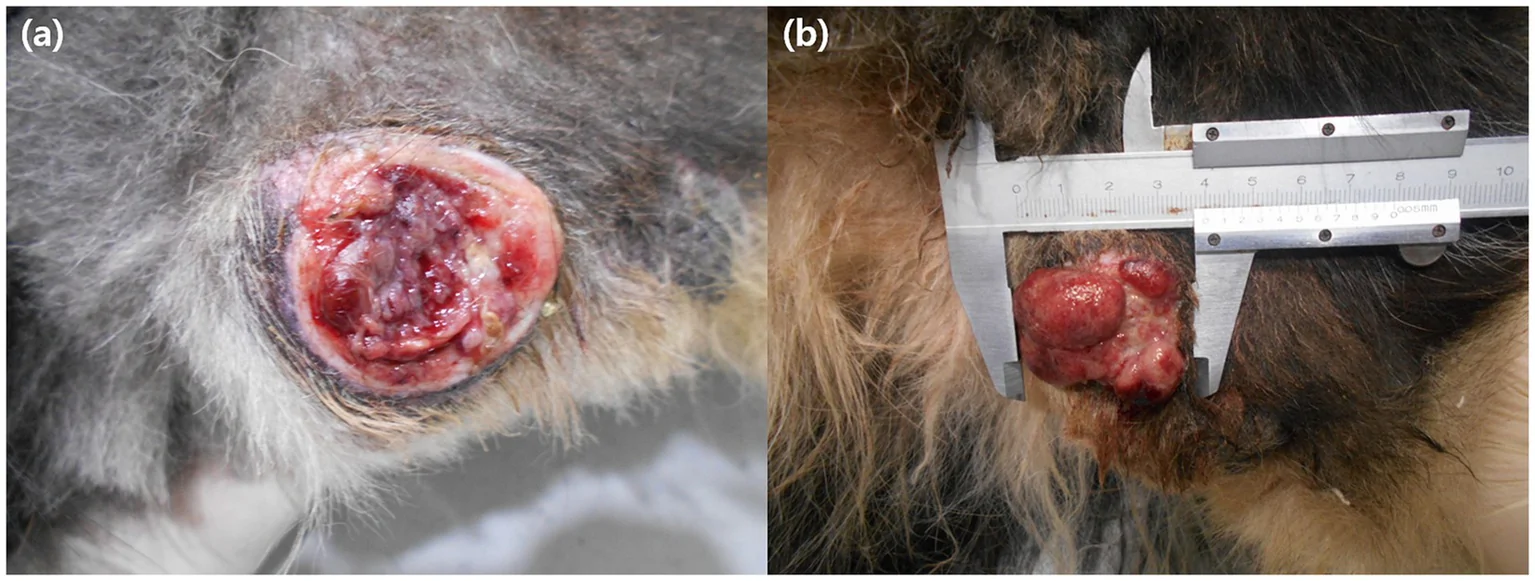
Clinical progression of the right elbow lesion in the same dog. **(a)** At initial presentation (Day 0), a hemorrhagic and ulcerative lesion was observed on the right elbow. **(b)** Nine days after initial presentation (Day 9), the same lesion showed rapid progression, with an increase in size and a more prominent protruding mass measuring 3.7 × 2.5 cm **(b)**.

**Figure 2:**
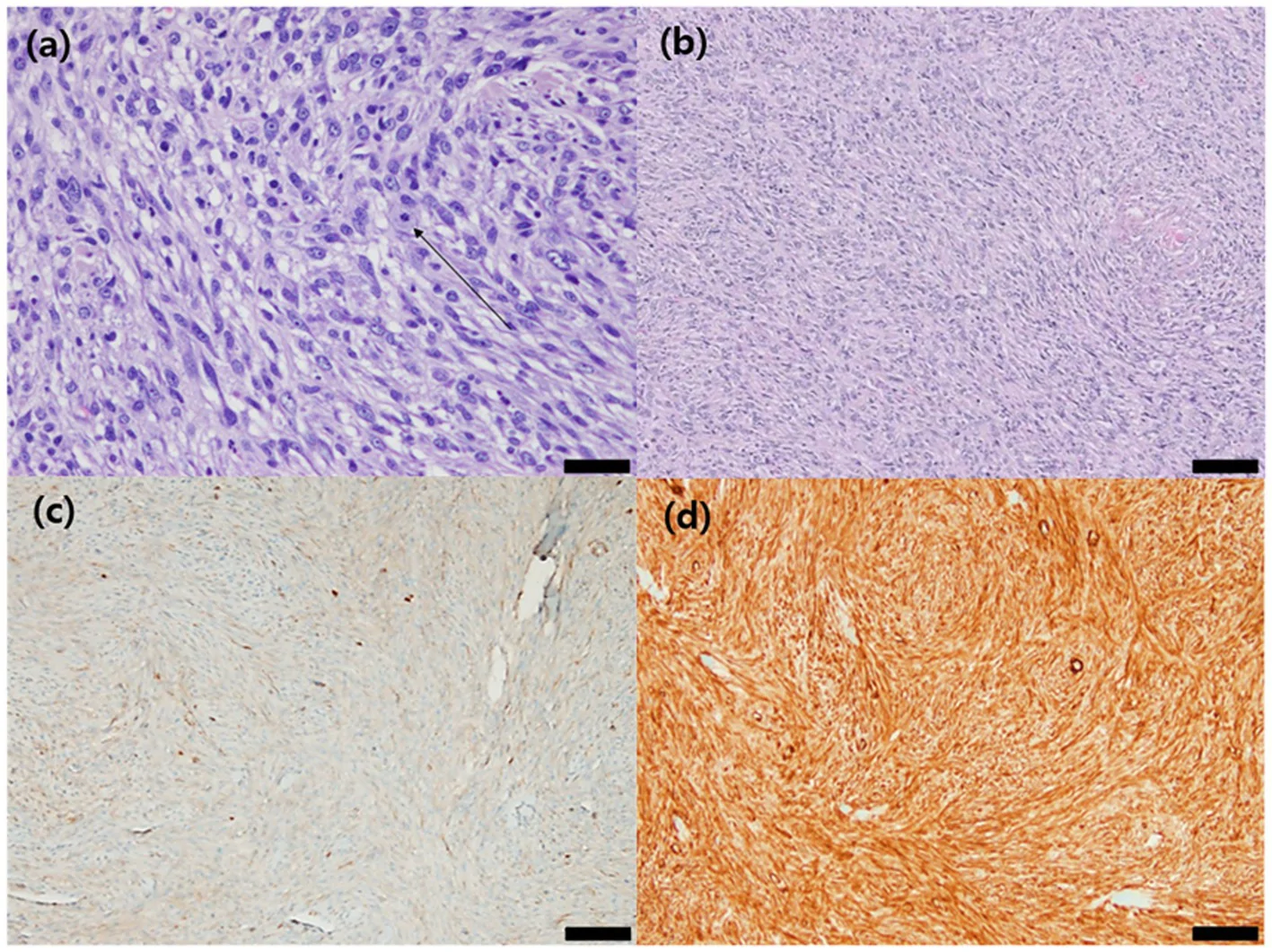
Histopathological and immunohistochemical findings of the preoperative biopsy and surgical excision specimens. Preoperative biopsy showed interlacing fascicles of spindle-shaped cells with pleomorphic nuclei, with a mitotic figure (black arrow) of 5 per 10 high-power fields [**(a)** original magnification ×40, H&E stain, bar = 25 μm]. Postoperative histopathological examination of the surgical excision specimen revealed poorly differentiated, elongated mesenchymal cells with moderate eosinophilic cytoplasm arranged in interlacing fascicles [**(b)** original magnification ×10, H&E stain, bar = 100 μm]. Positive immunoreactivity for desmin [**(c)** original magnification ×10, bar = 100 μm] and strong positive immunoreactivity for alpha-smooth muscle actin [**(d)** original magnification ×10, bar = 100 μm] were identified.

Pre-anesthetic laboratory evaluation revealed mild neutrophilia, mild anemia, slight hyperglobulinemia, and markedly increased alkaline phosphate activity (Supplementary Table S2). Follow-up radiographs obtained prior to PET/CT demonstrated interval enlargement of the oval soft tissue mass to approximately 6.2 × 4.5 cm, without adjacent cortical bone lysis (Supplementary Figure S1b). Thoracic and abdominal radiographs were performed as part of the pre-anesthetic evaluation and metastatic screening, and revealed no evidence of pulmonary nodules or other metastatic lesions (Figure 3a).

**Figure 3:**
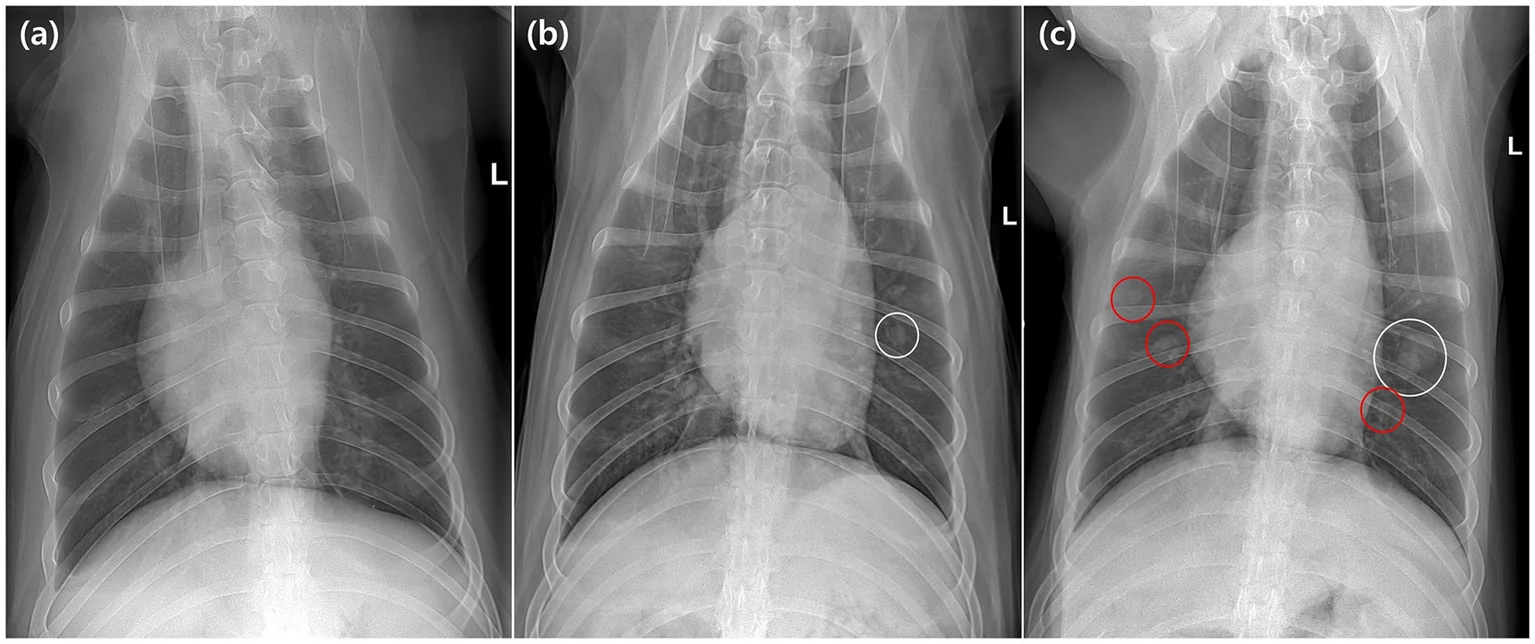
Serial thoracic radiographic images (ventrodorsal view). No evidence of pulmonary metastasis was identified prior to ^18^F-FDG PET/CT **(a)**. A nodular radiopaque lesion consistent with a pulmonary metastasis was identified in the right caudal lung field (white circle) 3 months postoperatively **(b)**. At recheck 3 weeks later, the existing nodule had increased in size (white circle) and additional pulmonary nodules (red circles) were newly identified, indicating rapid disease progression **(c)**. ^18^F-FDG, ^18^F-fluorodeoxyglucose; PET/CT, positron emission tomography/computed tomography.

For further evaluation of tumor malignancy and potential metastasis, PET/CT was subsequently performed under general anesthesia. The dog was fasted for 12 h prior to ^18^F-FDG administration. Premedication consisted of intravenous midazolam (0.2 mg/kg; Midazolam, Bukwang Pharm. Co., Ltd., Seoul, South Korea), followed by induction with propofol (8 mg/kg; Provive, Myungmoon Pharm. Co., Ltd., Seoul, South Korea). Anesthesia was maintained with isoflurane (Terrell, Piramal Critical Care, Bethlehem, PA, USA) during imaging. The dog was placed in sternal recumbency, and vital signs were monitored. Prior to the PET scan, CT images were obtained through Multi-detector CT scanner (Discovery-STE, General Electric Medical Systems, Waukesha, WI, USA) with 100 mAs, at 120 kVp in 1.25 mm slice thickness. After intravenous administration of 880 mg/kg iohexol (Omnipaque, GE Healthcare, Marlborough, MA, USA), contrast scans were performed. There was an ill-defined soft-tissue mass at the right elbow with heterogenous contrast enhancement in post-contrast images (Figures 4a,b).

**Figure 4:**
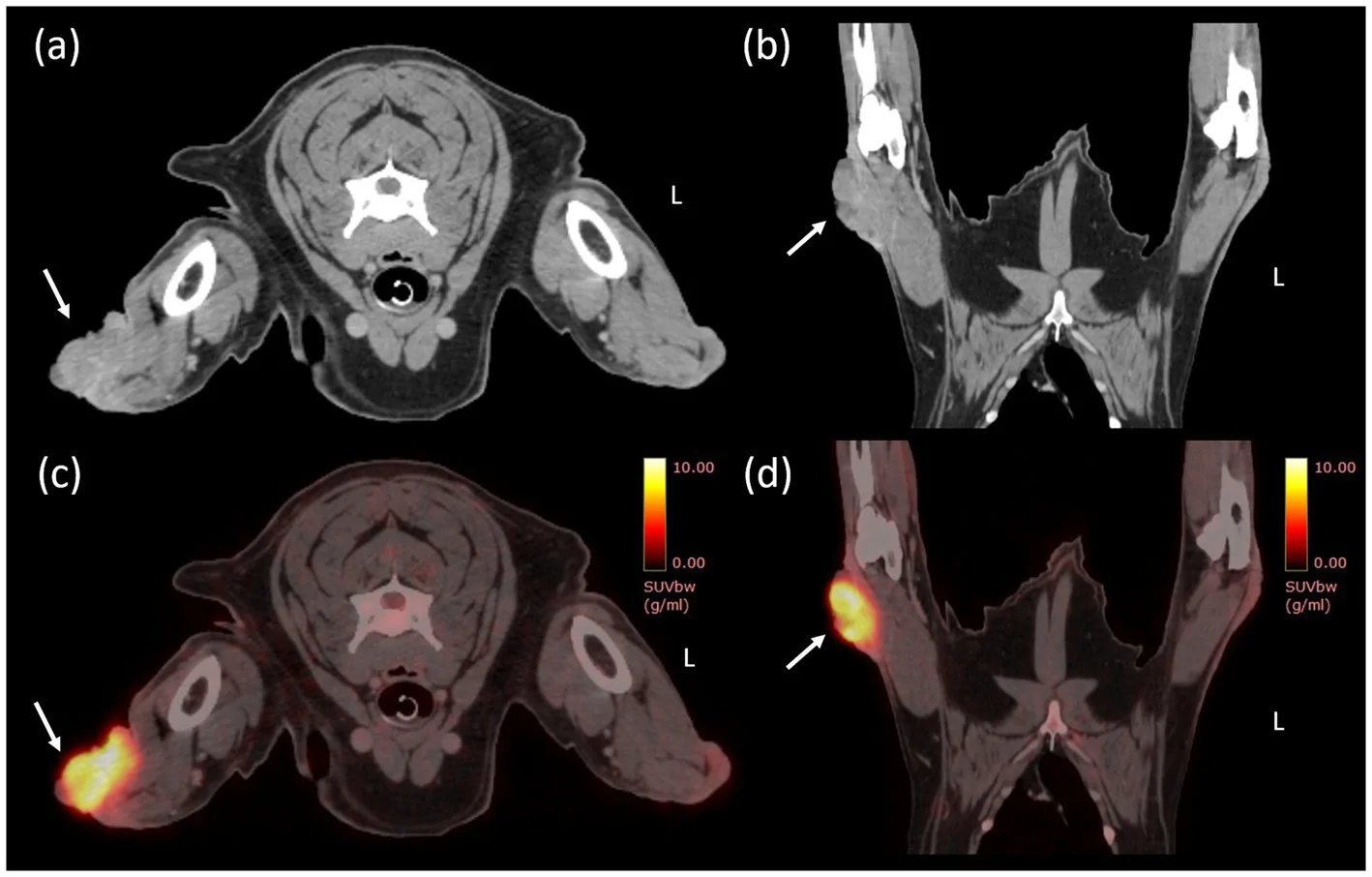
^18^F-FDG PET/CT findings in a dog with subcutaneous leiomyosarcoma. Transverse **(a)** and dorsal **(b)** post-contrast CT images demonstrate an ill-defined soft-tissue mass with heterogenous contrast enhancement (arrows) at the right elbow. Corresponding transverse **(c)** and dorsal **(d)** PET/CT fusion images show markedly increased ^18^F-FDG uptake in the mass (arrows), with an SUVmax of 10.1 and an SUVmean of 4.6. The color scale bar indicates the degree of ^18^F-FDG uptake. ^18^F-FDG, ^18^F-fluorodeoxyglucose; PET, positron emission tomography; CT, computed tomography; SUVmax, maximum standardized uptake value; SUVmean, mean standardized uptake value.

Subsequently, intravenous injection of ^18^F-FDG was administered at a dose of 239.02 MBq (6.29 MBq/kg) followed by a saline flush and 60 min of uptake period. PET image was analyzed by OsiriX MD (Pixmeo, Bernex, Switzerland). The standardized uptake value (SUV) quantifies tissue uptake of ^18^F-FDG (MBq/mL) by adjusting for the injected dose (MBq) and the individual’s body weight (kg). PET/CT scan showed increased ^18^F-FDG uptake in the right elbow mass, with maximum standardized uptake value (SUVmax) and mean standardized uptake value (SUVmean) values of 10.1 and 4.6, respectively (Figures 4c,d). No abnormal ^18^F-FDG avid lesion was detected in areas other than the tumor, including regional lymph nodes, and no evidence of metastasis was found. These ^18^F-FDG PET/CT findings suggested that the tumor was a highly metabolically active lesion with no evidence of other primary sites or distant metastasis, thereby supporting the diagnosis of a primary subcutaneous STS. Based on this interpretation, limb amputation was recommended because PET/CT findings suggested a highly aggressive malignant tumor. However, the owner declined amputation, and surgical resection of the primary lesion was performed 2 weeks later. The excised mass was subsequently submitted for definitive histopathological evaluation. Histopathological examination revealed a poorly differentiated mesenchymal neoplasm (differentiation score 3), with a mitotic count exceeding 40 per 10 high-power fields (mitotic score 3) and necrosis involving less than 50% of the sample (necrosis score 2) (Figure 2b). The summed score of 8 corresponded to a grade 3 STS (13). Excision was incomplete, with neoplastic cells extending to the deep surgical margin. To further determine the histologic origin of the tumor, immunohistochemistry (IHC) for desmin and alpha smooth muscle actin was performed, and the neoplastic cells showed positive immunoreactivity for both markers (Figures 2c,d). Based on the histopathological and IHC findings, the tumor was diagnosed as subcutaneous LMS, a distinct mesenchymal tumor, rather than a conventional STS subtype.

Following the histopathologic diagnosis of a high-grade tumor with incomplete surgical margins, limb amputation and adjuvant chemotherapy were recommended. However, the owner declined both treatment options, and oral prednisolone and analgesics were administered as palliative care. At the 1-month postoperative follow-up, thoracic radiographs revealed no evidence of pulmonary nodules or other metastatic lesions, while radiographs of the right elbow showed that the previously identified mass was no longer visible following surgical resection, with no evidence of adjacent bone lysis (Supplementary Figure S1c). Three months after surgery, the dog developed cough, and thoracic radiographs revealed a pulmonary nodule, indicating a potential for metastasis (Figure 3b). Local tumor recurrence was also observed, with progressive regrowth at the surgical site. Follow-up imaging performed 3 weeks after the initial detection of the pulmonary nodule showed an increase in both the size and number of the pulmonary nodules (Figure 3c). Given the progression of both local and pulmonary lesions, systemic chemotherapy was recommended again; however, the owner declined further diagnostic evaluations and treatment and opted to maintain palliative care. The dog expired 6 months post-surgery (7 months after the first visit) confirmed by a phone call with the owner.

## 3 Discussion

Prognosis in canine STS is closely linked to tumor grade, with higher-grade tumors exhibiting increased metastatic potential and shorter survival time (4). Grade 1 STS has a favorable prognosis, with a median survival exceeding 3 years after complete excision. Grade 2 STS has a median survival of 1–2 years, with a metastasis rate of up to 20%. Grade 3 STS is highly aggressive, with a median survival of less than 1 year and metastasis rates up to 50% (4, 14, 15). Surgical margins also play a key role in tumor control, as complete excision significantly reduces recurrence risk (4, 15). A study on subcutaneous STS found local recurrence rates of 7%, 34%, and 75% for grade 1, 2, and 3 tumors, respectively (15). Given the strong association between tumor grade and prognosis, accurate preoperative assessment is essential for guiding treatment decisions.

^18^F-FDG PET/CT has been used in human and veterinary oncology to assess tumor metabolism, malignancy grading, and prognosis (12, 16–19). In human STS, ^18^F-FDG PET/CT has demonstrated the ability to differentiate benign from malignant lesions, with malignant STS exhibiting an SUVmax range of 0.6–14 (mean: 5.9), compared with 1.2–6.2 (mean: 3.5) for benign lesion (16). An SUVmax threshold of 3.9 and an SUVmean of 2.5 have been proposed to distinguish grade 2–3 from grade 1 STS (17), and SUVmax of 1.3, 2.7, and 4.5 have been reported for grade 1, 2, and 3 STS, respectively (18). Variability in FDG uptake across histologic subtypes has also been reported (19). While ^18^F-FDG PET/CT has proven useful in STS evaluation in human medicine, its application in veterinary oncology remains limited, with few studies to date characterizing FDG uptake in canine STS (12). That study reported SUVmax ranging from 2.0 to 10.6 and SUVmean from 1.2 to 5.8, with the highest uptake observed in an undifferentiated STS (12); however, tumor grades were not documented, precluding direct comparisons with malignancy grading. In this case, ^18^F-FDG PET/CT demonstrated a highly metabolically active tumor with an SUVmax of 10.1 and an SUVmean of 4.6, exceeding reported ranges of SUVmax for grade 3 STS in humans (18). Although concurrent purulent inflammation may have contributed to the increased ^18^F-FDG uptake, inflammatory changes alone are unlikely to fully explain the marked metabolic activity observed in this case. Considering the final histopathologic diagnosis of a high-grade (grade 3) LMS, the PET/CT findings were considered to be more consistent with the biological behavior of the tumor than with inflammation alone. Notably, the high ^18^F-FDG uptake observed on PET/CT corresponded with the high histologic grade subsequently identified by postoperative histopathology. These findings support the role of ^18^F-FDG PET/CT as a tool complementary to conventional histopathology, providing metabolic insights that preoperative biopsy alone may not fully capture, and underscore its potential value in guiding treatment decisions in canine STS.

The confirmation of subcutaneous LMS in this case raises important considerations regarding its classification and prognostic implication. While LMS has historically been classified within the STS framework, accumulating evidence supports its recognition as a biologically distinct entity due to its unique histological features and clinical behavior (2, 4, 5). The prognosis of canine LMS varies considerably depending on the anatomical site of origin, histologic grade, and treatment approach (4, 5). Visceral LMS, commonly arising in the gastrointestinal tract, liver, or uterus, is associated with high metastatic potential and a poor long-term prognosis, with a reported median survival of approximately 10 months (ranging from 1 month to 7 years) (1). In contrast, non-visceral LMS, including cutaneous and subcutaneous forms, remains less well characterized in dogs, and its clinical behavior is poorly understood. In humans, subcutaneous LMS is considered a more aggressive entity than its cutaneous counterpart, demonstrating higher local recurrence rates and a poorer clinical course (20). Whether a similar biological distinction exists in dogs remains uncertain, as the available veterinary literature on non-visceral LMS consists mainly of limited case reports (3, 21), and comparative outcome studies between cutaneous and subcutaneous forms are lacking.

^18^F-FDG PET/CT has proven valuable in human LMS for staging, monitoring disease progression, and correlating SUV with tumor grade and prognosis (7–11). Higher SUVmax has been associated with increased tumor aggressiveness and poorer survival outcomes in human LMS, consistent with findings in other high-grade STS (10, 11, 18). Longitudinal PET/CT analyses further suggest that SUVmax tends to increase in recurrent human LMS, a pattern not consistently observed in other STS subtypes, indicating a potentially more aggressive recurrence behavior (22). Despite its established role in human oncology, ^18^F-FDG PET/CT studies in veterinary LMS remain scarce. To date, only one study has reported PET/CT findings in canine LMS – a case of gastric LMS histologically confirmed as grade 1, in which an SUVmax of 6.234 and an SUVmean of 4.596 were documented (23). In the present case, the subcutaneous LMS exhibited a substantially higher SUVmax of 10.1, which may reflect its higher histologic grade (grade 3) compared with the grade 1 gastric LMS previously reported. Additionally, given that skin metastasis from a visceral primary could not be clinically excluded at initial presentation, ^18^F-FDG PET/CT served to confirm the right elbow mass as the primary lesion with no evidence of a visceral source. Collectively, these findings suggest that ^18^F-FDG PET/CT may serve as a valuable tool for primary site evaluation, malignancy grading, and prognosis assessment in canine LMS, particularly in subcutaneous forms where clinical behavior remains poorly characterized.

Following surgical resection without adjuvant chemotherapy, the tumor progressed rapidly, with local recurrence and suspected pulmonary metastasis developing 3 months postoperatively; the dog expired 6 months after surgery. This clinical course is consistent with a poor prognosis for subcutaneous LMS and underscores the importance of a more aggressive therapeutic approach in cases with high histologic grade and incomplete resection margins. Given the correspondence between high FDG avidity and the high histologic grade observed in this case, ^18^F-FDG PET/CT may provide complementary information for preoperative tumor assessment, thereby guiding more proactive management strategies, including more aggressive surgical management (e.g., limb amputation when appropriate), adjuvant chemotherapy, and closer postoperative monitoring.

A limitation of this case is the non-specificity of ^18^F-FDG uptake, as active inflammation within the ulcerated tumor may have contributed to the high ^18^F-FDG uptake observed on PET/CT. Therefore, the relative contributions of tumor metabolism and inflammation could not be determined. Another limitation is that additional immunohistochemical markers, such as h-caldesmon or calponin, were not evaluated. Although these markers may provide further support for smooth muscle differentiation, the diagnosis was based on the histomorphologic findings together with diffuse immunoreactivity for desmin and α-smooth muscle actin (2, 3). In addition, Ki-67 immunostaining was not performed. Although Ki-67 may provide additional information regarding tumor proliferative activity, histologic grading was determined using conventional histopathologic criteria, including mitotic count. Additionally, pulmonary nodules suspected to represent metastatic lesions were not histologically or cytologically confirmed. Their progressive increase in size and number on serial thoracic radiographs was suggestive of pulmonary metastasis; however, a definitive diagnosis could not be established.

## Data Availability

The original contributions presented in the study are included in the article/Supplementary material, further inquiries can be directed to the corresponding author.
