# Supplementary material for: Case Report: 18F-FDG PET/CT for precision assessment of tumor aggressiveness in canine subcutaneous leiomyosarcoma
Source: Front Vet Sci. 2026 Oct 19;13:1948200. doi: 10.3389/fvets.2026.1948200 (PMC13760838; doi:10.3389/fvets.2026.1948200)
Supplement: Supplementary file 1 [file Table_1.DOCX]

**Supplementary Figure S1. Radiographic evaluation of the right elbow.**

Serial radiographs of the right elbow are shown in (a–c). Each panel includes mediolateral and craniocaudal views, with arrows in (a) and (b) indicating the soft tissue mass. (a) At the initial presentation, an approximately 4.3 × 3.1 cm oval soft tissue mass is visible caudal to the elbow joint, without adjacent bone lysis. (b) Approximately 1 month later, immediately prior to PET/CT, the mass measures approximately 6.2 × 4.5 cm, with no evidence of adjacent bone lysis. (c) At the 1-month postoperative follow-up, the previously identified mass is no longer visible following surgical resection, with no evidence of adjacent bone lysis. PET/CT, positron emission tomography/computed tomography.

**Supplementary Table S1. CARE-based timeline of the clinical course of the dog with subcutaneous leiomyosarcoma**

| **Time point** | **Key clinical events** |
| --- | --- |
| Day 0 | Initial presentation of a hemorrhagic, ulcerative right elbow lesion; cytology revealed atypical cells with concurrent inflammation and bacterial organisms; empirical antimicrobial treatment was initiated. |
| Day 9 | Rapid lesion progression; biopsy findings were consistent with a grade 2 STS. |
| Day 26 | ^18^F-FDG PET/CT demonstrated high FDG uptake in the right elbow mass (SUVmax, 10.1); with no evidence of distant metastasis. |
| Day 41 | Surgical resection; histopathology revealed a grade 3 STS with an incomplete deep surgical margin, and IHC confirmed subcutaneous LMS. |
| Day 72 | No pulmonary nodules were detected at the 1-month postoperative follow-up. |
| Day 131 | Local tumor recurrence and a pulmonary nodule were detected. |
| Day 152 | An increase in the size and number of pulmonary nodules was detected. |
| Day 221 | Death. |

Abbreviations: ¹⁸F-FDG, ¹⁸F-fluorodeoxyglucose; IHC, immunohistochemistry; LMS, leiomyosarcoma; PET/CT, positron emission tomography/computed tomography; STS, soft tissue sarcoma; SUVmax, maximum standardized uptake value.

**Supplementary Table S2. Pre-anesthetic laboratory findings.**

| **Parameter** | **Result** | **Reference interval** |
| --- | --- | --- |
| **Complete blood count** |  |  |
| WBC counts (×10^3^/μL) | 17.91 | 5.05–16.76 |
| Lymphocyte counts (×10^3^/μL) | 2.28 | 1.05–5.10 |
| Neutrophil counts (×10^3^/μL) | 14.19 | 2.95–11.64 |
| RBC counts (×10^6^/μL) | 4.96 | 5.65–8.87 |
| Hematocrit (%) | 29.8 | 37.3–61.7 |
| Platelet counts (×10^3^/μL) | 258 | 148–484 |
| **Serum biochemistry** |  |  |
| Albumin (g/dL) | 2.9 | 2.6–3.3 |
| Globulin (g/dL) | 4.5 | 2.7–4.4 |
| Alanine aminotransferase (IU/L) | 39 | 21–102 |
| Alkaline phosphatase (IU/L) | 626 | 29–97 |
| BUN (mg/dL) | 23 | 7–25 |
| Creatinine (mg/dL) | 0.6 | 0.5–1.5 |
| Glucose (mg/dL) | 115 | 65–118 |
